# Respiratory syncytial virus infection in children during SARS-CoV-2 pandemic at a referral center in Rio de Janeiro, Brazil

**DOI:** 10.36416/1806-3756/e20240072

**Published:** 2024-07-29

**Authors:** Giuliana Pucarelli Lebreiro, Marianna Tavares Venceslau, Maria Angélica Arpon Marandino Guimarães, Thalita Fernandes Abreu, Yarina Rangel, Ana Cristina Cisne Frota, Cristina Barroso Hofer

**Affiliations:** 1. Departamento de Doenças Infecciosas e Parasitárias, Universidade Federal do Rio de Janeiro - UFRJ - Rio de Janeiro (RJ) Brasil.; 2. Instituto de Puericultura e Pediatria Martagão Gesteira, Universidade Federal do Rio de Janeiro - UFRJ - Rio de Janeiro (RJ) Brasil.

**Keywords:** SARS-CoV-2, Respiratory syncytial virus infections, Pediatrics, Brazil

## Abstract

**Objective:**

In order to study the scenario of respiratory infections in pediatrics after the emergence of SARS-CoV-2 in Brazil, this study aimed to compare characteristics of children admitted for SARS or upper airway infection caused by either RSV or SARS-CoV-2.

**Methods:**

This was a cross-sectional study involving children up to 48 months of age admitted to a tertiary pediatric hospital with a diagnosis of SARS or upper airway infection between April of 2020 and April of 2021. Respiratory secretion samples were collected 2-5 days after hospitalization, and antigen/PCR tests for viral etiologies were performed. In this analysis, patients with laboratorial diagnosis of SARS-CoV-2 and/or RSV were selected, and their clinical and epidemiological characteristics were compared using logistic regression.

**Results:**

Our sample initially comprised 369 participants. SARS-CoV-2 and RSV infections were confirmed in 55 (15%) and 59 children (16%), respectively. Mean age was 12 months (0-48 months), and 47 were female. The following characteristics were significantly more frequent in patients with RSV when compared with those with COVID-19: younger age (OR = 0.94; 95% CI: 0.90-0.98); lower frequency of fever (OR = 0.18; 95% CI: 0.05-0.66); and more frequent upper airway symptoms: cough (OR = 7.36; 95% CI: 1.04-52.25); and tachypnea (OR = 6.06; 95% CI: 1.31-28.0).

**Conclusions:**

Children with RSV-related SARS were younger, had lower frequency of fever at admission, but had a higher frequency of signs of upper airway infection and lower systemic inflammation when compared with children hospitalized for COVID-19 during the first year of the pandemic.

## INTRODUCTION

Respiratory syncytial virus (RSV) is the most common lower respiratory infection etiology during the first years of life. Approximately 60% of infants are infected with RSV during their first six months of age, and 80% at their first birthday.^1^

The main clinical manifestations of RSV infection are mild and comprise fever, cough, and nasal discharge. However, around 20% of these will develop more critical disease, with lower respiratory tract manifestations such as tachypnea, lower oxygen saturation, and poor feeding. Some of these infants will require hospital admission to provide respiratory support.^2^

After 2019, a new etiologic agent was described causing respiratory disease in humans. Infection with SARS-CoV-2 is more severe in adults, showing higher mortality and case-fatality rates. This agent was also described as an important respiratory infection in children with several clinical manifestations, including bronchiolitis.^3^ ^,^ ^4^

Several authors described that the SARS-CoV-2 pandemic changed the epidemiological scenario of RSV infection in the pediatric population. A lower number of hospital admissions and differences on RSV seasonality were found.^2^ ^,^ ^5^ ^,^ ^6^

The clinical manifestations of RSV during the pandemic were also described as different from those in previous years, mainly because more patients were admitted with symptoms in the higher respiratory tract instead of in the lower respiratory tract.^5^ ^,^ ^7^

Since different approaches are recommended for different respiratory viruses, the etiology of respiratory tract infections must be determined. Molecular or antigen-based tests, with high sensitivity and specificity, are used; however, their costs are still a barrier in developing countries, where clinical and basic laboratory tests are the main diagnosis tools available.

In this study, we aimed to describe the main signs and symptoms in children with RSV infection admitted to a tertiary university-based hospital in the city of Rio de Janeiro, Brazil, during the first year of the SARS-CoV-2 pandemic and compare the clinical manifestations of RSV and SARS-CoV-2 infections in children up to 48 months of age.

## METHODS

### Study design and participants

This was a nested cross-sectional study, and data were originated from a prospective cohort study of children and adolescents hospitalized at a tertiary pediatric hospital: *Instituto de Puericultura e Pediatria Martagão Gesteira* (IPPMG) of the Federal University of Rio de Janeiro, which is a referral center for rheumatology, immunology, infectious diseases, and onco-hematology care in the city of Rio de Janeiro, Brazil. The children and adolescents who had suspected COVID-19 diagnosis between April 14, 2020 and April 30, 2021 were enrolled.^3^

All patients under 48 months of age admitted at our institution with upper airway infection (with or without fever) and/or SARS were eligible for inclusion. Exclusion criteria were being older than 48 months at admission or RT-PCR for SARS-CoV-2 results being unavailable.

### Data collection

Following enrollment, all parents or legal guardians of the participants completed an initial questionnaire, and the participants were followed during hospitalization for data collection.

The variables collected included age, gender, race, socioeconomic status, previous contact with a suspected or confirmed COVID-19 case, contact with indoor smoking, underlying medical conditions, signs and symptoms, oxygen requirement, admission to critical care, presence of other viral coinfections, and laboratory test results.

### Ethical aspects

Parents/guardians of participants provided written informed consent before participation. The study was reviewed and approved by the IPPMG Research Ethics Committee (no. 30786020.4.0000.5264). All clinical specimens were obtained in accordance with ethical guidelines.

### Definitions

A participant was considered as infected with SARS-CoV-2 when RT-PCR for SARS-CoV-2 was positive or undetermined from any clinical sample (nasopharyngeal or oropharyngeal swab, bronchoalveolar lavage, stool, or cerebrospinal fluid) and if serological results were positive (IgM and IgG or IgG) 30 days after the onset of symptoms. We consider reagent IgG as a confirmatory laboratory criterion only in unvaccinated individuals, with no previous laboratory diagnosis for COVID-19 and who presented compatible signs and symptoms within the first six months of the study (and the first six months of the pandemic). Patients with positive RT-PCR for SARS-CoV-2 and positive serological tests were compared with those with positive RSV tests. The date of symptom onset was defined as the day when the first symptom or sign occurred.

### Sample collection and viral detection

The specimens collected varied according to the patient’s symptoms. Nasopharyngeal and nasal swabs specimens were collected with synthetic fiber swabs; each swab was inserted into a separate sterile tube containing 2 mL of phosphate buffered saline. In intubated or tracheostomized patients, tracheal aspirate specimens were obtained the same way. Specimens were immediately processed at the Virology Laboratory or stored at 4°C until processing. Nucleic acid extraction was performed manually with the Bio Gene Viral DNA/RNA extraction kit (Bioclin, Belo Horizonte, Brazil). SARS-CoV-2 RNA was detected using both the CDC 2019-nCoV RT-PCR Diagnostic Panel and Bio-Manguinhos SARS-CoV-2 Molecular Kit using the 7500 Real-Time PCR System (Applied Biosystems, Waltham, MA, USA). The Charité/Berlin protocol was used, and samples were considered SARS-CoV-2 positive when both E and RdRP target genes were detected, and if the CT value was not higher than 37.

Additional diagnostic tests were performed for children aged 0-3 months (multiplex respiratory PCR panel; FilmArray; BioFire Diagnostics, Salt Lake City, UT, USA) and those aged 3-48 months (Influenza and RSV PCR tests). SARS-CoV-2 serology was performed using a chemiluminescence immunoassay (Kit MAGLUMI 2019-nCOV IgG/IgM CLIA; Snibe, Shenzhen, China). All assays were performed according to manufacturer instructions. Tests were performed according to hospital unit availability for testing. For this study, we selected participants who had a confirmed diagnosis of either COVID-19 or RSV (i.e., cases of coinfection were not included in this analysis).

### Statistical analyses

The data collected was compiled using Microsoft Access for Windows 10, version 2019. All variable distributions were studied. Categorical variables were described as absolute and relative frequencies; continuous variable distributions were studied graphically and were described as means and standard deviations. Bivariate analysis was performed using the Fisher’s exact test (categorical variables) or the Mann-Whitney test (continuous variables). Independent variables with p < 0.2 were included in a multivariate analysis model, using backward stepwise logistic regression. Variables with < 5% of frequency were not added into the multivariate analysis model. Since this study took place in a tertiary hospital, it was common that patients with comorbidities were admitted to the emergency room, and these comorbidities might have had a role in the pathogenicity of the respiratory infections in study; therefore, we forced this variable (presence or not of a comorbidity) into the final model. The main dependent variable was a diagnosis of SARS-CoV-2 or RSV infection. Models with and without interactions were tested using the −2 log-likelihood test. The fitness of the model was evaluated using the Hosmer-Lemeshow test. Alternative hypotheses were accepted if the comparison p-value was ≤ 0.05. All data were analyzed using the Stata software, version 15 (Stata Corp LP, College Station, TX, USA).

## RESULTS

In this analysis, among 369 patients eligible for the study, we detected 114 patients infected with either SARS-CoV-2 (n = 59; 52%) or RSV (n = 55; 48%) during the first year of the COVID-19 pandemic. The mean age was 12 months (range: 0-48 months).

The time distribution of RSV cases during the first year of the SARS-CoV-2 pandemic was different from their normal distribution. Most of the cases (75%) occurred between Brazilian late spring and summer (between October of 2020 and January of 2021), whereas they regularly occur between late autumn and winter (from May to August).

RSV patients were younger than were patients with SARS-CoV-2 infection. No differences were found regarding gender or other sociodemographic variables between the groups. RSV patients presented with respiratory distress manifestations, while patients with SARS-CoV-2 presented with more systemic and inflammatory manifestations (fever at admission and higher inflammatory markers, such as protein C reactive or white blood cells; Table 1).

**Table 1:** Comparison of clinical manifestations and laboratory test results between patients infected with RSV or SARS-CoV-2 (bivariate analysis).^a^

Variable	RSV N = 59 (52%)	SARS-CoV-2 N = 55 (48%)	p*
Age, months	7.9	20.1	< 0.01
Gender, female	26 (44.1)	21 (40.3)	0.42
Family income^b^	1.6	1,3	0.07
Tobacco exposure at household	21(37.5)	14 (28)	0.31
Fever at admission	32 (54)	41 (75)	0.01
Cough	57 (97)	35 (64)	< 0.01
Rhinorrhea	43 (75)	26 (49)	< 0.01
Tachypnea	54 (93)	29 (53)	< 0.01
Dyspnea	49 (88)	25 (48)	< 0.01
SpO_2_ < 95%	30 (53)	13 (23)	< 0.01
Rash	2 (3.5)	5 (9.4)	0.26
Abdominal pain	24 (24.9)	10 (18.9)	< 0.01
Diarrhea	14 (24.6)	18 (32.7)	0.35
Vomiting	18 (32.1)	23 (41.8)	0,33
Chronic disease	19 (33.9)	32 (61.5)	< 0.01
Hemoglobin, g/dL	11.0	10.7	0.39
White blood cells, cells/mm^3^	10,956	12,845	0.20
Lymphocytes, cells/mm^3^	5202	4092	0.02
Platelets, count/mm^3^	356,707	350,816	0.83
Protein C reactive mg/dL	16.0	54.1	< 0.01

a Values expressed as mean or n (%). ^b^Brazilian minimum salary = US$300 *Variables with p < 0.05 were included in the initial multivariate analysis.

When we consider the multivariate analysis, none of the laboratory testing variables was significant. Children infected with RSV tended to be younger and presented with a phenotype of respiratory distress at admission, whereas those infected with SARS-CoV-2 tended to present with a systemic disease (fever or higher inflammatory enzymes; Table 2).

**Table 2:** Comparison of clinical manifestations between patients infected with RSV or SARS-CoV-2 (multivariate analysis).

Variable	OR	95% CI	p
Age, months	0.94	0.90-0.98	< 0.01
Fever at admission	0.18	0.05-0.66	< 0.01
Rhinorrhea	2.33	0.64- 8.46	0.19
Tachypnea	6.06	1.31-28.01	0.02
Chronic disease	0.52	0.17-1.63	0.26
Cough	7.36	1.04-52.25	0.04

None of the participants died. Admissions to the ICU in the SARS-CoV-2 and RSV groups were 6/55 (11%) and 13/59 (22%), respectively (p = 0.11). The mean length of hospital stay was seven days in both groups (p = 0.77).

## DISCUSSION

This prospective, nested cross-sectional study compared clinical and laboratory data of children aged 0-4 years infected with either RSV or SARS-CoV-2 during the first year of the COVID-19 pandemic. The time distribution of RSV cases was studied during this period.

During the study year, there was a time shift of RSV cases, from winter to summer. The main clinical manifestations of RSV cases were as a respiratory disease in younger children, while SARS-CoV-2 infection was more frequent in older children, who presented with high fever.

The distribution of the RSV cases during the first year of the SARS-CoV-2 pandemic was different from that observed in the southeast region of Brazil in previous years, during which most cases occurred during autumn and winter (May to August). In 2020/2021, most of the cases were in the summer. This phenomenon was also observed in other countries in the southern hemisphere, such as Australia and Thailand.^5^ ^,^ ^6^ We believe that the prolonged social isolation that occurred in our country in the first months of the pandemic was responsible for the deviation of the curve of RSV cases during summer.

Although SARS-CoV-2 is a new respiratory disease etiology, its phenotype is different from the RSV-related bronchiolitis in children up to four years of age, even when controlling for chronic diseases.

A Thai study that compared RSV cases before and during pandemic found that the cases that required hospitalization during the pandemic were less severe when compared with those before the pandemic.^5^ Although it was not the main aim of this study, we found no differences regarding the description of RSV phenotype.

In an Australian surveillance study of the RSV cases during the first year of the pandemic,^6^ the authors observed that RSV patients were older than in previous years (on average, 18 months). In our series of RSV cases, the children were younger (mean age, 8 months) and even younger than the children with SARS-CoV-2 infection.

A study also comparing SARS-CoV-2 and RSV cases in children up to two years of age in Italy described the same results as ours; children with the former infection had more fever, but the latter etiologic agent was more severe, although the authors did not adjust their findings for age and/or chronic diseases, as we did in our study.^8^

Pediatricians are used to diagnosing RSV infections based on the clinical manifestations of this infection in young populations^1^ ^,^ ^2^; however, after the introduction of a new agent in the differential diagnosis of respiratory diseases in children, it is important to understand the differences between these major agents. Some studies carried out in high-income countries made comparisons using small sample sizes,^6^ ^-^ ^8^ leaving an information gap regarding children in middle- and low-income countries such as Brazil.^9^

An important limitation in our study was that the data derived from the first year of the COVID-19 pandemic; since then, several SARS-CoV-2 variants have circulated in Brazil, and some of these presented with different clinical manifestations when compared with others. Comparisons between clinical manifestations and variants over time in children demonstrated that some manifestations were more frequent for each variant, but disease severity was the same.^10^

In this study, we were able to demonstrate that the seasonality of RSV infection during the first year of the SARS-CoV-2 pandemic shifted from winter to summer, as described in other countries in the southern hemisphere.

The clinical phenotypes of the two different etiologies are different, and the differences pointed out in this study are important for pediatricians working at emergency departments in middle- and lower-income countries, where diagnosis tests and therapies are limited.

## References

[1] Bardsley M, Morbey RA, Hughes HE, Beck CR, Watson CH, Zhao H (2023). Epidemiology of respiratory syncytial virus in children younger than 5 years in England during the COVID-19 pandemic, measured by laboratory, clinical, and syndromic surveillance a retrospective observational study. Lancet Infect Dis.

[2] Jiang MY, Duan YP, Tong XL, Huang QR, Jia MM, Yang WZ (2023). Clinical manifestations of respiratory syncytial virus infection and the risk of wheezing and recurrent wheezing illness a systematic review and meta-analysis. World J Pediatr.

[3] National Institutes of Health [homepage on the Internet] (c2023). COVID-19 Treatment Guidelines: Therapeutic Management of Hospitalized Children With COVID-19.

[4] Pucarelli-Lebreiro G, Venceslau MT, Cordeiro CC, Maciel FQ, Anachoreta TD, de Abreu TF (2022). Clinical Manifestations and Complications of Children With COVID-19 Compared to Other Respiratory Viral Infections A Cohort Inpatient Study From Rio de Janeiro, Brazil. Front Pediatr.

[5] Chaiut W, Sapbamrer R, Dacha S, Sudjaritruk T, Parwati I, Sumarpo A (2023). Characteristics of Respiratory Syncytial Virus Infection in Hospitalized Children Before and During the COVID-19 Pandemic in Thailand. J Prev Med Public Health.

[6] Foley DA, Yeoh DK, Minney-Smith CA, Martin AC, Mace AO, Sikazwe CT (2021). The Interseasonal Resurgence of Respiratory Syncytial Virus in Australian Children Following the Reduction of Coronavirus Disease 2019-Related Public Health Measures. Clin Infect Dis.

[7] Ozeki S, Kawada JI, Yamashita D, Yasufuku C, Akano T, Kato M (2022). Impact of the Coronavirus Disease 2019 Pandemic on the Clinical Features of Pediatric Respiratory Syncytial Virus Infection in Japan. Open Forum Infect Dis.

[8] Pruccoli G, Castagno E, Raffaldi I, Denina M, Barisone E, Baroero L (2023). The Importance of RSV Epidemiological Surveillance A Multicenter Observational Study of RSV Infection during the COVID-19 Pandemic. Viruses.

[9] Freitas AR, Donalisio MR (2016). Respiratory syncytial virus seasonality in Brazil implications for the immunisation policy for at-risk populations. Mem Inst Oswaldo Cruz.

[10] Sumner MW, Xie J, Zemek R, Winston K, Freire G, Burstein B (2023). Comparison of Symptoms Associated With SARS-CoV-2 Variants Among Children in Canada. JAMA Netw Open.

